# Metagenomic Detection of RNA Viruses of *Hyalomma asiaticum* Ticks in the Southern Regions of Kazakhstan

**DOI:** 10.3390/microorganisms13092064

**Published:** 2025-09-05

**Authors:** Kulyaisan T. Sultankulova, Nurlan S. Kozhabergenov, Gaukhar O. Shynybekova, Olga V. Chervyakova, Bekbolat S. Usserbayev, Dana A. Alibekova, Asankadir T. Zhunushov, Mukhit B. Orynbayev

**Affiliations:** 1 Research Institute for Biological Safety Problems, National Holding QazBioPharm, The Ministry of Health Care of the Republic of Kazakhstan, Gvardeiskiy 080409, Kazakhstan; 2 Institute of Biotechnology, National Academy of Science of Kyrgyzstan, Bishkek 720071, Kyrgyzstan

**Keywords:** tick, metagenomics, virome, RNA, virus, *Hyalomma asiaticum*, Kazakhstan

## Abstract

*Hyalomma* ticks are known for their ability to transmit a wide range of pathogens, posing a significant threat to both human and animal health. The viral communities associated with *Hyalomma asiaticum* ticks in Kazakhstan remain largely unexplored. Using high-throughput sequencing on the Ion Torrent platform, nine RNA viruses belonging to seven families were identified. These viruses were associated with *H. asiaticum* ticks collected in 2024 in southern Kazakhstan. The detected viruses—including Bole tick virus 1 (*Phenuiviridae*), Bole tick virus 3 (*Chuviridae*), Bole tick virus 4 (*Flaviviridae*), Hubei toti-like virus 24 (*Totiviridae*), Kashgar totiv tick virus 1 (*Totiviridae*), Lonestar tick totivirus (*Totiviridae*), Qingyuan parti tick virus 1 (*Partitiviridae*), and Taishun tick virus (*Rhabdoviridae*)—had previously been reported only in China. This study provides the first documented evidence of the presence of RNA viruses previously unreported in Kazakhstan within *H. asiaticum* tick populations. A newly isolated Kazakhstan strain of Wad Medani virus (*Sedoreoviridae*), identified from *H. asiaticum*, shares genetic similarities with Russian strains, suggesting a common epidemiological landscape across Central Eurasia. The detection of novel RNA viruses in Kazakhstan highlights the need for ongoing surveillance, as their impact on human and animal health remains insufficiently understood.

## 1. Introduction

Tick-borne diseases represent a serious and often underestimated threat to global public health [1]. Ticks exhibit susceptibility to diverse environmental conditions, enabling their survival across a wide range of ecosystems, including extreme desert and semi-desert regions [2]. Central Asia is one of the world’s most vulnerable regions to intensive desertification, with over 60% of its territory classified as arid land [3]. In Kazakhstan, approximately 66% of the land is affected by desertification. The most affected areas include the desiccated Aral Sea basin in the Kyzylorda region and the arid border zones in the Zhambyl and Turkestan regions. Desertification directly impacts biodiversity, creating unique dryland ecosystems inhabited by organisms adapted to such conditions, including ticks—major vectors of various pathogens. In these environments, tick populations can increase, leading to elevated epidemiological risks for humans and livestock.

The genus *Hyalomma* includes 27 species, primarily distributed in the Afrotropical region and parts of the Palearctic region [4]. The species *H. asiaticum* is widespread in several Asian countries, including Afghanistan, Armenia, Azerbaijan, China, Georgia, Iran, Iraq, Kazakhstan, Kyrgyzstan, Mongolia, Pakistan, Russia, Syria, Tajikistan, Turkey, Turkmenistan, and Uzbekistan [5,6]. Several viruses of medical or veterinary significance have been identified in association with *H. asiaticum* ticks, such as Wad Medani virus (WMV, *Sedoreoviridae*) [7], Syr-Darya Valley fever virus (SDVFV, *Picornaviridae*) [8], and Tamdy virus (TAMV, *Nairoviridae*) [9]. Wad Medani virus has been found in ticks and can cause fever in humans and, in some cases, myocarditis or hemorrhagic manifestations. Syr-Darya Valley fever virus causes an acute febrile illness with chills, headache, and rash. Tamdy virus affects humans, rodents, and domestic animals, causing fever, weakness, myalgia, and, in some cases, transient neurological symptoms. This species is also a known vector of high-risk pathogens, including Crimean-Congo hemorrhagic fever virus (CCHFV, *Nairoviridae*) [10,11]. CCHFV is classified as a highly dangerous infection, with potential use as a bioterrorism agent and a case fatality rate ranging from 3% to 30% in humans [12].

The current epidemiological situation is characterized by a rise in known tick-borne infections and the emergence of novel pathogens. Many natural foci overlap geographically, meaning a single tick species can transmit multiple pathogens simultaneously. This phenomenon leads to mixed infections, which pose significant challenges for clinical diagnosis, treatment, and prevention and thus require integrated scientific investigation. Ticks have become such efficient vectors of infectious agents that several tick-borne viral diseases are now recognized as emerging global threats [13].

Similar ecosystems have developed in the border regions between Kazakhstan and China, supporting shared tick species such as *Hyalomma*, *Dermacentor*, and *Ixodes*. These ticks harbor infectious agents of veterinary and zoonotic concern in both countries [14]. With the rapid advancement of next-generation sequencing (NGS) technologies in recent years, numerous novel viral sequences have been identified in ticks from diverse regions of the world [15].

Based on this background, the aim of this study was to conduct a metagenomic analysis of *H. asiaticum* tick samples to identify and characterize their virome. We collected ticks from ecosystems in southern Kazakhstan and performed metagenomic sequencing to investigate the diversity of RNA viruses associated with *H. asiaticum*. This study represents the first targeted virome analysis of *H. asiaticum* ticks in Kazakhstan.

## 2. Materials and Methods

### 2.1. Tick Collection and Sample Processing

Tick samples were collected from three southern regions of Kazakhstan: Kyzylorda, Turkestan, and Zhambyl. Ticks were manually removed from cattle using forceps during the period from April to October 2024.

Samples were transported to the laboratory on the same day whenever possible. If immediate transport was not feasible, adult ticks were stored in plastic tubes containing grass and kept in a cool environment or refrigerated for up to ten days. Each tube was labeled with detailed information, including the collection date, tick species, number of individuals, and sampling location.

**Figure 1:**
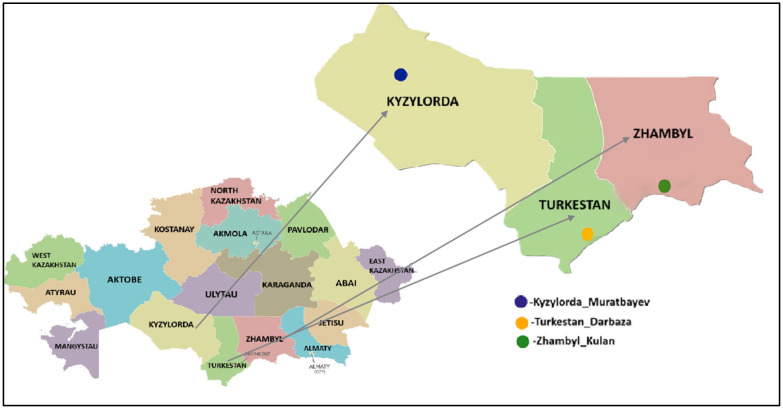
Geographic locations of *H. asiaticum* tick collection sites in southern Kazakhstan. Sampling was conducted in three regions: Kyzylorda (Muratbayev village), Turkestan (Darbaza village), and Zhambyl (Kulan village). The geographic locations of *H. asiaticum* tick collection sites were mapped using QGIS software version 3.34.

Live ticks were delivered to the laboratory and thoroughly sterilized by rinsing twice with 75% ethanol for 30 s. After sterilization, the samples were stored at −80 °C until further processing. The geographical location of the *H. asiaticum* tick collection sites is shown in Figure 1.

### 2.2. Tick Identification

Ticks were identified morphologically under a stereomicroscope (RS0745, Altami, Saint Petersburg, Russia), following standard taxonomic keys [16], and subsequently confirmed by molecular genetic analysis based on the mitochondrial cytochrome c oxidase subunit 1 (COX1) gene [17]. The COX1 gene was amplified using the primers Cox1F: 5′-GGAACAATATATTTAATTTTTGG-3′ and Cox1R: 5′-ATCTATCCCTACTGTAAATATATG-3′ (820 bp). PCR amplification was performed under the following conditions: initial denaturation at 94 °C for 2 min; followed by 35 cycles of denaturation at 94 °C for 30 s, annealing at 54 °C for 45 s, and extension at 72 °C for 1 min; with a final extension step at 72 °C for 10 min.

PCR products of the COX1 gene were sequenced using the BigDye Terminator v3.1 Cycle Sequencing Kit (Applied Biosystems, Inc., Vilnius, Lithuania) on an Applied Biosystems 3130 genetic analyzer (HITACHI, Tokyo, Japan).

The COX1 reference sequences from the *Hyalomma* ticks used in this study are available in GenBank: PQ554938, KU880599, PX097067, MW498397, MN892553, NC062166, ON482186, KM235712, MN9078737, OM743220, KP792585, OP183495, MN511174, LC779159, and MT230045.

The GenBank accession numbers for the COX1 sequences of *H. asiaticum* ticks from Kazakhstan are PV687729, PV687724, and PV688046. Table 1 summarizes information on *H. asiaticum* tick samples collected from three regions of southern Kazakhstan: Kyzylorda (Muratbayev village), Turkestan (Darbaza village), and Zhambyl (Kulan village). For each sample, the following data are provided: sample ID, region, collection site, number of ticks analyzed (15 individuals per pool), tick species and sex, and host.

### 2.3. Viral RNA Extraction and cDNA Synthesis

Viral RNA was extracted from 9 pooled tick samples using the PureLink Viral RNA/DNA Mini Kit (Invitrogen, Thermo Fisher Scientific, Carlsbad, CA, USA). Each pool consisted of 15 ticks of the same species and sex, collected from a single location.

RNA concentrations were measured using a NanoDrop 2000 spectrophotometer (Thermo Fisher Scientific, Waltham, MA, USA). To assess the purity and quality of the extracted nucleic acids, the A260/A280 optical density ratio was used as an indicator of sample purity.

Complementary DNA (cDNA) was synthesized using the Ion Torrent NGS Reverse Transcription (RT) Kit (Thermo Fisher Scientific, Carlsbad, CA, USA).

### 2.4. NGS Library Preparation and Data Processing

For NGS library preparation, 30 µL of viral cDNA at a concentration of 10–100 ng/µL was used. Libraries were prepared by fragmentation and ligation using the Ion Plus Fragment Library Kit (Thermo Fisher Scientific, Carlsbad, CA, USA), and purification steps were performed with Agencourt AMPure XP magnetic beads (Beckman Coulter, Brea, CA, USA) according to the manufacturer’s instructions.

Horizontal gel electrophoresis was performed in 1.5% agarose gel (Sigma-Aldrich, Burlington, MA, USA), stained with SYBR™ Safe DNA Gel Stain (Invitrogen, Thermo Fisher Scientific, Carlsbad, CA, USA), in Tris-acetate buffer at 100 V/cm for 30 min. DNA visualization and documentation were conducted using the Invitrogen iBright CL1500 transilluminator (Thermo Fisher Scientific, Marsiling Industrial Estate, Woodlands, Singapore). A 1 kb DNA Ladder (Thermo Fisher Scientific, Carlsbad, CA, USA) was used as a molecular weight marker.

DNA fragments were excised from the gel and purified using the innuPREP DOUBLEpure Kit (Innuscreen, Berlin, Germany) according to the manufacturer’s protocol. Amplification of DNA libraries was performed using reagents from the Ion Plus Fragment Library Kit (Thermo Fisher Scientific, Carlsbad, CA, USA) following the manufacturer’s protocol.

After purification, the amplified libraries were quantified using the Ion Universal Library Quantitation Kit (Thermo Fisher Scientific, Carlsbad, CA, USA). Final libraries were prepared using the Ion Chef Instrument with the Ion 510, 520 & 530 Kit (Thermo Fisher Scientific, Marsiling Industrial Estate, Woodlands, Singapore) and loaded onto an Ion 530 Chip (Thermo Fisher Scientific, Carlsbad, CA, USA).

High-throughput sequencing of viral genomes was performed using the Ion Torrent platform on the Ion GeneStudio S5 sequencer (Thermo Fisher Scientific, Marsiling Industrial Estate, Woodlands, Singapore). Sequencing results were obtained using Ion Torrent Suite Software v5.12 in UBAM and FASTQ formats.

The quality and trimming of raw reads were assessed using FastQC (version 0.12.0). Quality-filtered reads were assembled using BWA-MEM (bwa-0.7.19), with input files in FASTQ format. Taxonomic classification was conducted using Kaiju software (version 1.10.1) and the nr_euk reference database [18].

### 2.5. Phylogenetic Analysis

To construct the phylogenetic tree, a set of nucleotide sequences was obtained from the international GenBank database (NCBI). The analysis included available nucleotide sequences of the following viruses from GenBank: Taishun tick virus: OP313016, OP313017, PP473532, PP945090, PV093845, PP473531, MH688523, KM817643, PV791384, MH688522, MN542372; Bole Tick Virus 1: PP945221, PP945219, MZ244252, PP945225, PP473520, PP945223, ON812175, KM817664, MH688502, MH688500; Lonestar tick totivirus: PP926299; Hubei toti-like virus: NC032938, KX882977; Kashgar totiv tick virus 1: PP926298, ON746541; Wad Medani virus: MF521575, MH571969, MG770345, MK978734, KP268812, NC027546; Bole tick virus 3: OP313023, MZ244264, PP473528, PP473525, MH688551, MH688555, KM817593, NC028259, MH688554, PV791382, MH688550; Bole tick virus 4: NC028371, KR902736, PV791383, MK774653, MH688540, MZ567076, PP945235, PP473510, MW561135, MW561976, MN095535.

In the Kazakhstani isolates of Bole tick virus 1, Lonestar tick totivirus, and Bole tick virus 3, a fragment of the gene encoding RNA-dependent RNA polymerase (RdRp) was sequenced. In the Taishun tick virus isolate, a fragment of the nucleocapsid protein gene (N-gene) was determined. In Bole tick virus 4, a fragment of the gene encoding polyprotein protein was sequenced. In Wad Medani virus, a fragment of the VP6 (helicase) gene was determined. In Kashgar totiv tick virus 1, a fragment of the structural protein gene was determined, and in Hubei toti-like virus 24, a fragment of the hypothetical protein 2 gene was determined.

BLASTn results for each tick-borne virus were compared with nucleotide sequences of relevant reference viruses selected for subsequent phylogenetic analysis. Multiple sequence alignments were performed using ClustalW in MEGA version 11.0 [19].

Phylogenetic trees were constructed using the Neighbor-Joining (NJ) and BioNJ algorithms based on a matrix of pairwise distances estimated using the Tamura–Nei model, followed by selection of the topology with the highest log-likelihood value. Evolutionary analyses were carried out in MEGA 11.0 [19,20].

### 2.6. Statistical Analysis

Statistical analyses were performed using R v4.0.2, integrated within RStudio v1.3.1093, and visualized using the ggplot2 package [21]. Alpha diversity was assessed using the Kruskal–Wallis rank sum test.

Comparative analysis of viral distribution across regions was also conducted using the Kruskal–Wallis test. To visualize overlaps in viral distribution, Venn diagrams were generated, where each area represents unique or shared viruses among regions, with statistically significant *p*-values indicated (*p* < 0.05) [22,23].

To quantitatively assess the diversity of the virome, three alpha diversity indices were calculated: Simpson [24], Shannon [25], and Pielou [26].

## 3. Results

### 3.1. Molecular Genetic Identification of Ticks

Tick samples were collected from cattle between April and October 2024. *H. asiaticum* ticks were transported to the laboratory under ambient environmental conditions. A total of 135 adult *H. asiaticum* ticks, randomly selected from those isolated from cattle in three regions of southern Kazakhstan, were used for virome analysis. Female tick samples were pooled in groups of 15 based on location, species, and sex. Additional information, such as sampling location and number of ticks, is provided in Table 1.

**Figure 2:**
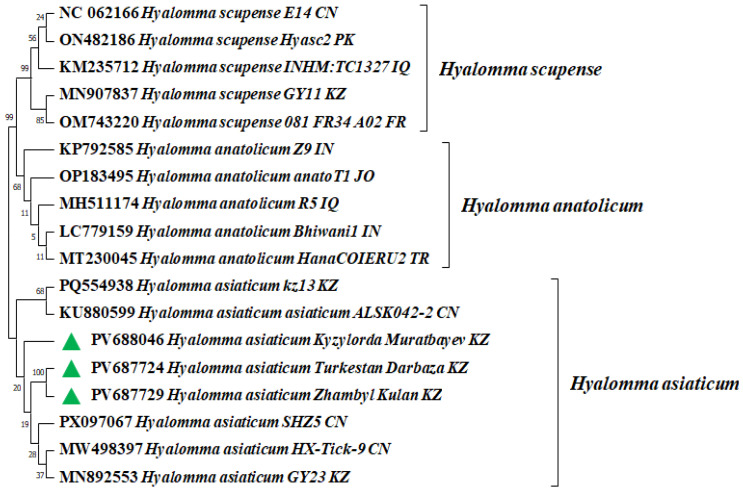
Phylogenetic analysis of the COX1 gene of *H. asiaticum* ticks identified in this study. 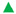: *H. asiaticum* identified in this study based on the COX1 gene. COX1 gene sequences were deposited in GenBank under the following accession numbers: PV687729—*Hyalomma asiaticum Zhambyl_Kulan_KZ*; PV687724—*Hyalomma asiaticum Turkestan_Darbaza_KZ*; PV688046—*Hyalomma asiaticum Kyzylorda_Muratbayev_KZ*. Phylogenetic analysis was performed using the Neighbor-Joining (NJ) and BioNJ methods based on the matrix of genetic distances calculated using the Tamura–Nei model.

The results of the molecular genetic identification of ticks based on the COX1 gene are shown in Figure 2.

Phylogenetic analysis of the COX1 gene sequences showed that *Hyalomma asiaticum Turkestan_Darbaza_KZ*, PV687724 and *Hyalomma asiaticum Zhambyl_Kulan_KZ*, PV687729 form a cluster with Kazakhstan (*Hyalomma asiaticum kz13 KZ*, PQ554938; *Hyalomma asiaticum GY23 KZ*, MN892553) and Chinese tick populations (*Hyalomma asiaticum asiaticum ALSK042-2 CN*, KU880599; *Hyalomma asiaticum SHZ5 CN*, PX097067; *Hyalomma asiaticum HX-Tick-9 CN*, MW498397), demonstrating high nucleotide identity within 99.82–100%.

At the same time, *Hyalomma asiaticum Kyzylorda_Muratbaev_KZ*, PV688046 also clusters with the mentioned Kazakh and Chinese tick populations, showing 100% identity with *Hyalomma asiaticum kz13 KZ*, PQ554938; *Hyalomma asiaticum GY2 KZ*, MN892553; *Hyalomma asiaticum asiaticum ALSK042-2 CN*, KU880599; *Hyalomma asiaticum SHZ5 CN*, PX097067 and *Hyalomma asiaticum HX-Tick-9 CN*, MW498397. This confirms the close genetic similarity of *H. asiaticum* populations in Kazakhstan and China.

Comparative analysis of the nucleotide sequences of the COX1 gene of three samples—*Hyalomma asiaticum Turkestan_Darbaza_KZ*, *Hyalomma asiaticum Zhambyl_Kulan_KZ*, and *Hyalomma asiaticum Kyzylorda_Muratbaev_KZ*—showed that they have 100% identity with each other, which indicates their complete genetic similarity.

### 3.2. Detection of Tick-Borne Viruses in H. asiaticum

Using metagenomic analysis, several tick-borne viruses were identified in *H. asiaticum* ticks collected in Kazakhstan. These included the Kazakhstan isolates of Bole tick virus 1 (*Phenuiviridae*), Bole tick virus 3, Bole tick virus 4 (*Flaviviridae*), Hubei toti-like virus 24 (*Totiviridae*), Kashgar totiv tick virus 1 (*Totiviridae*), Lonestar tick totivirus (*Totiviridae*), Taishun tick virus (*Rhabdoviridae*), Wad Medani virus (*Sedoreoviridae*), and Qingyuan parti tick virus 1 (*Partitiviridae*).

**Figure 3:**
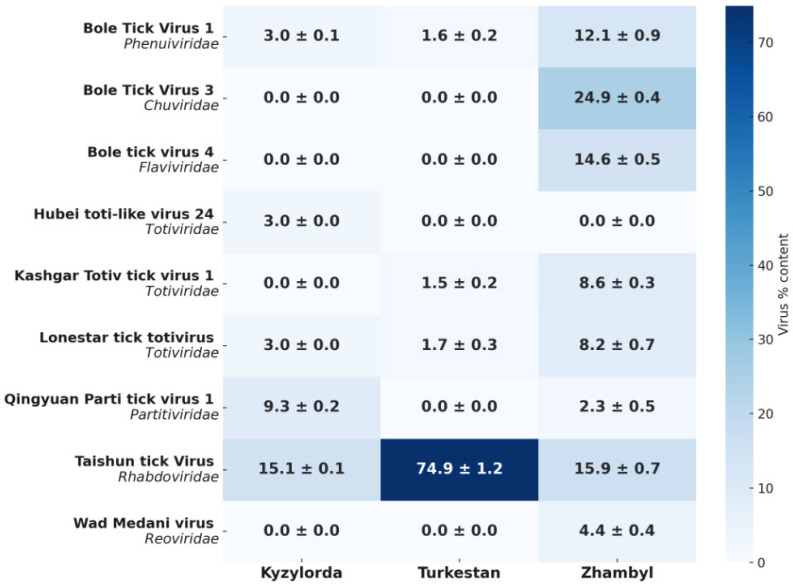
Relative abundance (%) of viruses detected in *H. asiaticum* ticks from southern Kazakhstan.

Differences were identified in the viral communities of *H. asiaticum* ticks collected from three southern regions of Kazakhstan: Kyzylorda, Turkestan, and Zhambyl. Figure 3 shows the relative abundance (%) of RNA viruses belonging to seven viral families.

Figure 3 shows the percentage distribution of each virus by region based on metagenomic data. The color scale indicates the relative abundance of tick-borne viruses. The relative abundance of a virus in a region is defined as the proportion of reads assigned to a given virus out of the total number of all viral reads in the pool, rather than out of all RNA, which also includes tick RNA. The heat map shows the distribution of viral taxa as a percentage within the viral pool based on metagenomic analysis, where each value represents the proportion of reads of a specific virus out of the total number of viral reads.

Analysis of viral communities revealed differences in the distribution of viruses between the three regions. The Zhambyl region had the highest diversity of viruses, including Bole tick virus 1, Bole tick virus 3, Bole tick virus 4, Taishun tick virus, Kashgar totiv tick virus 1, Lonestar tick totivirus, Qingyuan parti tick virus 1, and Wad Medani virus. The content of viruses in the region varied from ~2% to 25%; thus, there is no dominance of one taxon.

In the Turkestan region, the viral profile was significantly less diverse and was characterized by the predominance of Taishun tick virus, where it constituted ~75% of the total viral composition. The remaining viruses—Bole tick virus 1, Kashgar totiv tick virus 1 and Lonestar tick totivirus—were present only in small quantities (about 1.5–1.7%). In the Kyzylorda region, Taishun tick virus was detected, which dominated the composition of viruses, but its share was lower than in the neighboring region (15.1%). Qingyuan parti tick virus 1 was also detected here—9.3%. In addition, Bole tick virus 1, Hubei toti-like virus 24 and Lonestar tick totivirus were present in the region—each with a share of 3%.

**Figure 4:**
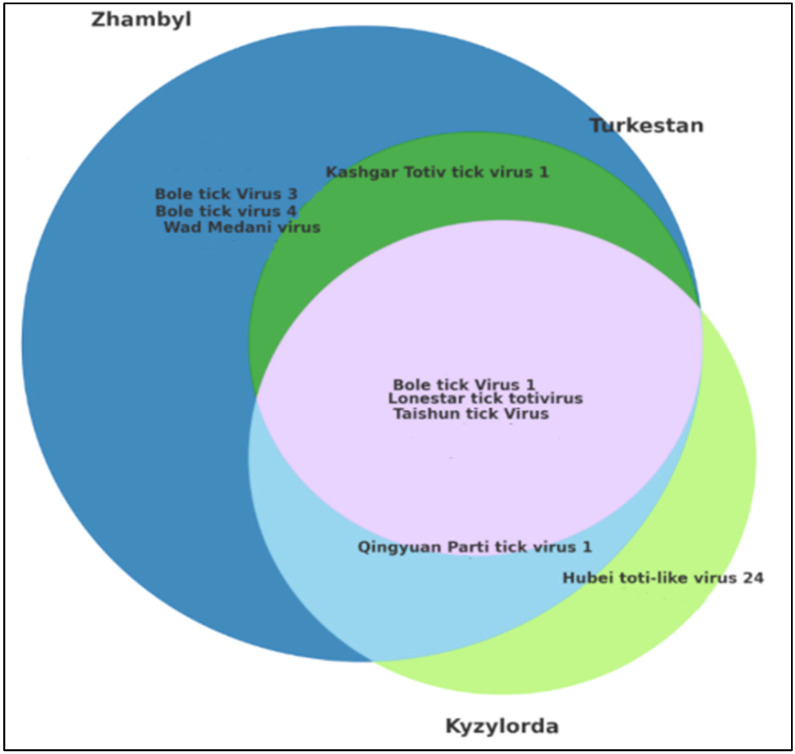
Venn diagram showing the regional distribution of tick-borne viruses along with *p*-values. 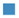: Bole tick virus 3, Bole tick virus 4, and Wad Medani virus were unique to the Zhambyl region; 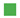: Kashgar totiv tick virus 1 was shared between the Zhambyl and Turkestan regions; 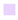: Bole tick virus 1, Lonestar tick totivirus, and Taishun tick virus were common to the Kyzylorda, Zhambyl, and Turkestan regions; 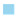: Qingyuan parti tick virus 1 was shared between the Zhambyl and Kyzylorda regions; 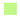: Wad Medani virus was uniquely detected in the Kyzylorda region.

The Venn diagram illustrates the overlap in the viral composition among the studied regions—Kyzylorda, Turkestan, and Zhambyl. This analysis enables the identification of region-specific viruses and the assessment of shared viruses across regions (Figure 4).

The circles in the Venn diagram represent viruses detected in each region, while the overlapping areas indicate the co-occurrence of the same viruses in two or more regions.

The overlapping zones correspond to the Kyzylorda, Turkestan, and Zhambyl regions. Non-overlapping sectors indicate viruses unique to a specific region. Analysis of the Venn diagram confirms the existence of both a shared viral core and region-specific viral patterns, which are important to consider when organizing epidemiological surveillance and studying the ecology of virus distribution.

Among the studied regions, the Zhambyl region demonstrated the highest viral diversity. Several viruses were detected exclusively in this region, indicating the presence of a unique tick virome. Viruses found only in Zhambyl included Bole tick virus 1, Bole tick virus 3, Bole tick virus 4, Taishun tick virus, Kashgar totiv tick virus 1, Lonestar tick totivirus, Qingyuan parti tick virus 1, and Wad Medani virus. This may be attributed to the ecological characteristics of the region or to differences in the composition of local *H. asiaticum* tick populations.

The Turkestan region was characterized by the presence of Lonestar tick totivirus, Kashgar totiv tick virus 1, Bole tick virus 1, and Taishun tick virus.

The Kyzylorda region exhibited a limited but unique viral profile. Hubei toti-like virus 24 was detected exclusively in this region. Other viruses identified in Kyzylorda included Bole tick virus 1, Taishun tick virus, Lonestar tick totivirus, and Qingyuan parti tick virus 1.

Notably, Bole tick virus 1, Lonestar tick totivirus, and Taishun tick virus were detected in all three regions, indicating their widespread distribution and stable transmission within *H. asiaticum* tick populations.

### 3.3. Alpha Diversity of H. asiaticum Virome

**Figure 5:**
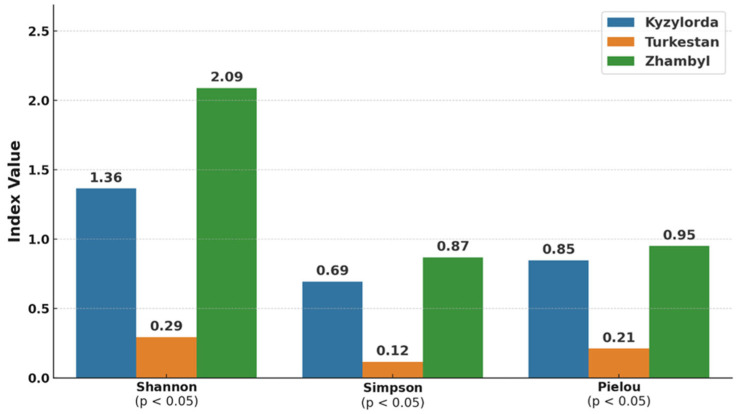
Alpha diversity of *H. asiaticum* viromes detected in the Kyzylorda, Turkestan, and Zhambyl regions based on Shannon, Simpson, and Pielou indices (blue—Kyzylorda region, orange—Turkestan region, green—Zhambyl region).

Analysis of alpha diversity of viruses detected in *H. asiaticum* ticks from three regions of Kazakhstan revealed significant regional differences based on the Shannon (H′), Simpson (D), and Pielou’s evenness (J′) indices. Statistical analysis using the non-parametric Kruskal–Wallis test showed significant differences among the regions for all three indices (*p* < 0.05) (Figure 5).

The Zhambyl region exhibited the highest values across all diversity indices: H′ = 2.09, D = 0.87, J′ = 0.95, reflecting a broad spectrum of viruses. Intermediate index values were observed in the Kyzylorda region: H′ = 1.36, D = 0.69, J′ = 0.85.

In contrast, the Turkestan region showed the lowest alpha diversity: H′ = 0.29, D = 0.12, J′ = 0.21, indicating a reduced viral diversity.

The greatest viral diversity and evenness were observed in the Zhambyl region, whereas the Turkestan region exhibited lower viral diversity due to the limited number of taxa and the dominance of a single virus.

### 3.4. Phylogenetic Analysis of Novel Tick-Borne Virus Isolates

Metagenomic sequencing enabled the identification and GenBank deposition of Kazakhstan isolates of the following viruses: Bole tick virus 3 (PV791382, SRR34988298), Bole tick virus 4 (PV791383, SRR34968736), Lonestar tick totivirus (PX125593, SRR34999688), and Taishun tick virus (PV791384, SRR34988297). A single consensus viral sequence from each tick pool was used to construct phylogenetic trees.

**Figure 6:**
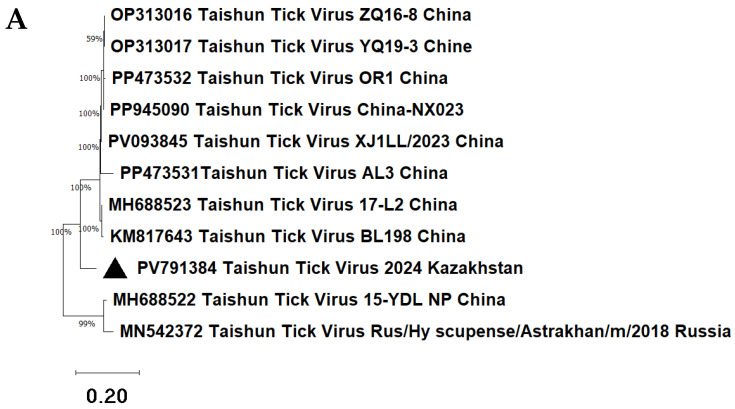

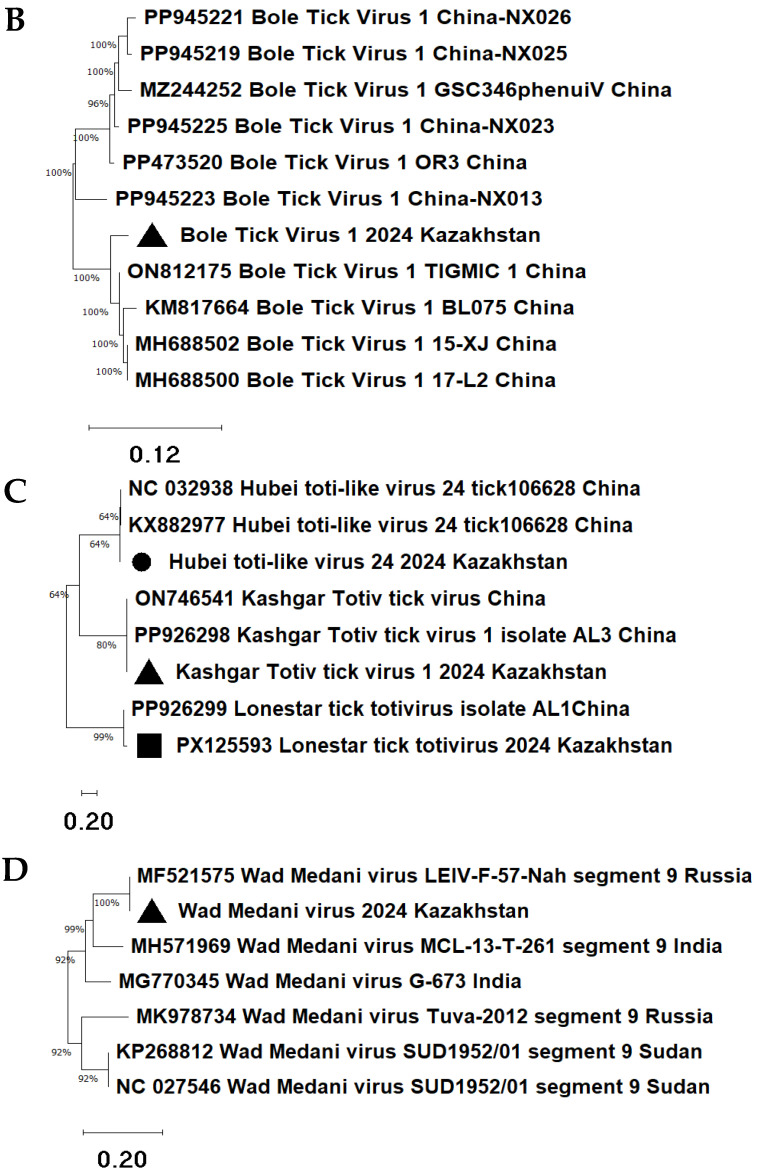

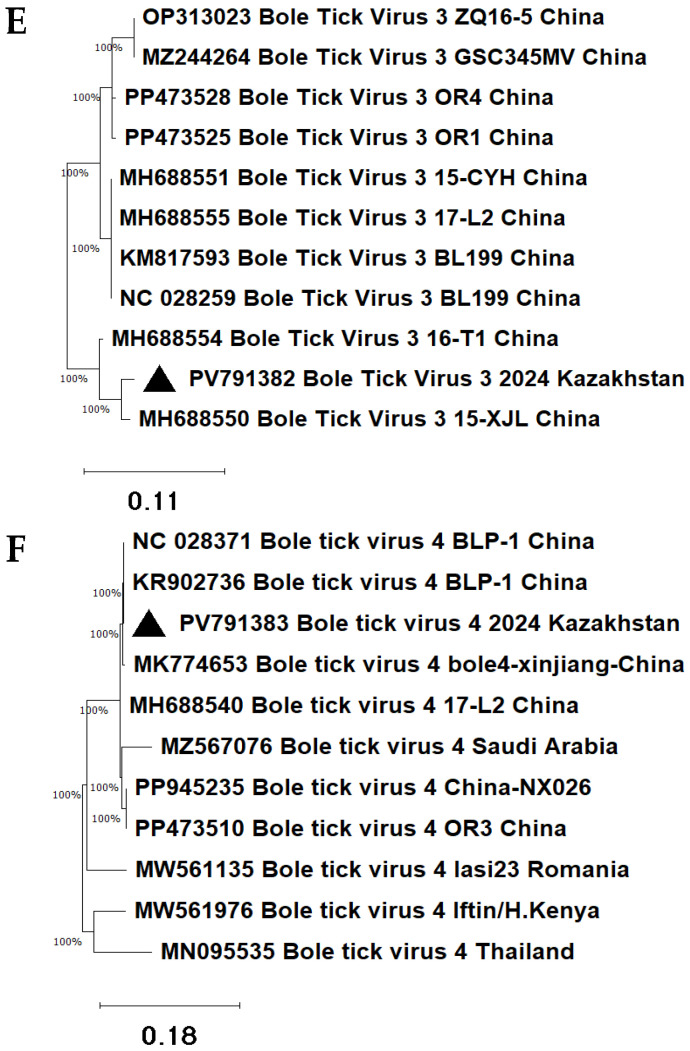
Phylogenetic analysis of viruses detected in *H. asiaticum* ticks collected in Kazakhstan in 2024. The phylogenetic trees were constructed using the Neighbor-Joining (NJ) and BioNJ algorithms based on a matrix of pairwise distances estimated using the Tamura–Nei model. Virus isolates from Kazakhstan are marked with black triangles on the panels. Only the isolates of Hubei toti-like virus 24 and Lonestar tick totivirus are marked with a black circle and a black square, respectively. (**A**) Fragment of the gene encoding the nucleocapsid of the isolate Taishun tick virus 2024 Kazakhstan; (**B**) fragment of the gene encoding the RNA-dependent RNA polymerase of the isolate Bole tick virus 1 2024 Kazakhstan; (**C**) fragment of the gene encoding the Hypothetical protein 2 of the isolate Hubei toti-like virus 24 2024 Kazakhstan; fragment of the gene encoding the structural protein of the isolate Kashgar totiv tick virus 1 2024 Kazakhstan; fragment of the gene encoding the RNA-dependent RNA polymerase of the isolate Lonestar tick totivirus 2024 Kazakhstan; (**D**) fragment of the gene encoding the VP6 (Hel) of the isolate Wad Medani virus 2024 Kazakhstan; (**E**) fragment of the gene encoding the RNA-dependent RNA polymerase of the isolate Bole tick virus 3 2024 Kazakhstan; (**F**) fragment of the gene encoding the polyprotein of the isolate Bole tick virus 4 2024 Kazakhstan.

In addition, partial nucleotide fragments were obtained for five other viruses: Bole tick virus 1, Hubei toti-like virus 24, Kashgar totiv tick virus 1, Wad Medani virus, and Qingyuan parti tick virus 1. Due to their limited length and fragmentation, these sequences were not submitted to GenBank. However, they were included in the phylogenetic analysis, as they showed high similarity to reference viral nucleotide sequences according to BLASTn results. The BLASTn analysis yielded highly significant E-values (E-value < 1 × 10^−50^), indicating a high degree of sequence homology based on NCBI criteria [27]. For each virus, nucleotide fragments of the genome were isolated. The lengths of the virus fragments were as follows: Hubei toti-like virus 24—137 nucleotides, Kashgar totiv tick virus 1—174, Lonestar tick totivirus—211, Bole tick virus 1—252, Bole tick virus 3—289, Bole tick virus 4—342 and Qingyuan parti tick virus 1—256 nucleotides, Wad Medani virus—127 nucleotides, Taishun tick virus—215 nucleotides. Fragments of viral genomes representing 9 RNA viruses from 7 families—Bole tick virus 1 (*Phenuiviridae*), Bole tick virus 3 (*Chuviridae*), Bole tick virus 4 (*Flaviviridae*), Hubei toti-like virus 24, Kashgar totiv tick virus 1, and Lonestar tick totivirus (*Totiviridae*), Qingyuan parti tick virus 1 (*Partitiviridae*), Taishun tick virus (*Rhabdoviridae*), and Wad Medani virus (*Sedoreoviridae*)—were analyzed phylogenetically to further characterize the tick-borne viral species circulating in *H. asiaticum* (Figure 6).

Phylogenetic analysis allowed the determination of the evolutionary origins of nine viral isolates detected in *H. asiaticum* in 2024.

The Taishun tick virus 2024 Kazakhstan isolate forms a cluster with a group of Taishun tick virus strains from China (Figure 6A). The sequence identity ranged from 86.86% (E-value = 4 × 10^−125^) to 89.43% (E-value = 9 × 10^−142^).

The Bole tick virus 1 isolate collected in 2024 is phylogenetically related to the Chinese strain Bole tick virus 1 TIGMIC 1 (ON812175) (Figure 6B), but it is placed in a separate subclade. The sequence similarity was 97.62% (E-value = 7 × 10^−117^).

Metagenomic analysis revealed three totivirus isolates of *H. asiaticum* from Kazakhstan: Kashgar totiv tick virus 1 2024 Kazakhstan, Hubei toti-like virus 24 2024 Kazakhstan, and Lonestar tick totivirus 2024 Kazakhstan (Figure 6C).

The Kashgar totiv tick virus 1 2024 Kazakhstan isolate clustered with the Chinese strain Kashgar totiv tick virus 1 AL3 (PP926298) and showed a sequence similarity of 99.42% (E-value = 6 × 10^−81^). Minor branching differences in the phylogenetic tree suggest regional divergence due to local viral evolution.

The Hubei toti-like virus 24 2024 Kazakhstan isolate was phylogenetically related to the Chinese strain Hubei toti-like virus 24 106628 (KX882977), but formed a separate subgroup, indicating genetic divergence. The level of similarity between the viruses was 98.54% (E-value = 7 × 10^−60^).

The Lonestar tick totivirus 2024 Kazakhstan isolate grouped with the strain Lonestar tick totivirus AL1 (PP926299), with 100% identity (E-value = 1 × 10^−145^).

The Wad Medani virus 2024 Kazakhstan isolate clustered with the Russian strain Wad Medani virus strain LEIV-F-57-Nah (MF521575), while Indian strains MH571969 and MG770345 were located on a more distant branch (Figure 6D). This indicates a 100% genetic similarity (E-value = 9 × 10^−58^) between viruses circulating in Kazakhstan and Russia, suggesting a shared epidemiological distribution across Central Eurasia.

The novel Bole tick virus 3 2024 Kazakhstan isolate clustered with the Chinese strain Bole tick virus 3 15-XJL (MH688550), indicating a phylogenetic relationship (Figure 6E). The identity level was 98.27% (E-value = 5 × 10^−139^).

The Qingyuan parti tick virus 1 2024 Kazakhstan isolate showed 91.80% identity (E-value = 5 × 10^−94^) with the Chinese isolate Qingyuan parti tick virus 1 isolate TIGMIC 1 (ON746478). The match was observed in a fragment of the gene encoding a hypothetical protein. No other published strains or genetic sequences in the NCBI database showed significant homology. Therefore, a complete phylogenetic tree could not be constructed.

The novel Bole tick virus 4 2024 Kazakhstan isolate clustered with the Chinese strain Bole tick virus 4 BLP-1 (NC028371). Both viruses were located on the same branch of the phylogenetic tree (Figure 6F). The identity level was 100% (E-value = 9 × 10^−177^). The Bole tick virus 4 2024 Kazakhstan isolate differs from viruses previously detected in other regions of China, the Middle East, Eastern Europe, and Southeast Asia.

## 4. Discussion

*H. asiaticum* ticks are important ectoparasites of livestock in Central Asian countries, particularly in desert, semi-desert, and steppe regions where they are widely distributed. Adult ticks parasitize cattle and may also bite humans. Due to their strong adaptation to arid climates and their capacity for intensive blood-feeding, *H. asiaticum* is considered an effective vector of viral pathogens [28]. Particular attention is paid to *H. asiaticum* due to its role in the transmission of Crimean-Congo hemorrhagic fever virus (CCHFV)—one of the region’s key arboviruses [10,29]. The widespread distribution of this tick species has been confirmed in both China [30] and Kazakhstan [31], suggesting the potential for transboundary transmission of tick-borne viruses between these countries.

The study included the southern regions of Kazakhstan (Zhambyl, Turkestan, and Kyzylorda regions) due to the known prevalence of *H. asiaticum* ticks in these areas, as well as registered cases of tick-borne infections in humans and animals. These regions are of interest for epidemiological monitoring, since they have previously been practically not studied from the standpoint of metagenomic analysis of tick viruses.

In this work, the main emphasis was placed on the qualitative identification of viruses and their phylogenetic characteristics. In the future, upon obtaining full-length genomes, it will be possible to conduct a more in-depth comparative genetic analysis, including an assessment of coverage, sequencing depth, nucleotide divergence, and interrelated phylogenetic relationships.

Metagenomic analysis of *H. asiaticum* ticks from southern Kazakhstan revealed nine viruses belonging to seven families: *Phenuiviridae, Chuviridae, Flaviviridae, Rhabdoviridae, Sedoreoviridae, Totiviridae,* and *Partitiviridae*. In particular, three viruses—Bole tick virus 1 (*Phenuiviridae*), Lonestar tick totivirus (*Totiviridae*), and Taishun tick virus (*Rhabdoviridae*)—were detected in all three regions: Kyzylorda, Turkestan, and Zhambyl. This suggests their wide circulation within *H. asiaticum* populations and highlights the tick’s role as both a reservoir and vector of viruses. The studied viruses belong to different genera: Wad Medani virus is classified in the genus *Orbivirus* (*Sedoreoviridae*), Taishun tick virus belongs to the genus *Alpharicinrhavirus* (*Rhabdoviridae*), Tamdy virus belongs to the genus *Orthonairovirus* (*Nairoviridae*), Bole tick virus 3 belongs to the genus *Mivirus* (*Chuviridae*), and Bole tick virus 1 belongs to the genus *Phlebovirus* (*Phenuiviridae*). The generic affiliation of Hubei toti-like virus 24, Kashgar totiv tick virus 1, Lonestar tick totivirus, Bole tick virus 4, and Qingyuan parti tick virus 1 remains unclear, which is being worked out at the current stage.

Other tick species are known to carry certain tick-borne viruses. For example, Bole tick virus 4 and Hubei toti-like virus 24 were previously isolated from *Dermacentor silvarum* and *Ixodes persulcatus*, respectively [32]; Lonestar tick totivirus was found in *Haemaphysalis concinna* [32]; and Taishun tick virus in *Haemaphysalis hystricis* [33]. These findings demonstrate the capacity of viruses to adapt to diverse vectors and facilitate their geographic spread.

Phylogenetic analysis revealed the diversity of viruses circulating in *H. asiaticum* in Kazakhstan, including both strains closely related to those found in China and unique local isolates. For instance, the Kazakhstan Taishun tick virus isolate clustered with a Chinese strain, confirming their genetic relatedness. Since this virus belongs to the *Rhabdoviridae* family [15], its zoonotic potential cannot be ruled out, especially in the event of mutations.

The Kazakhstan totiviruses—Kashgar totiv tick virus 1, Hubei toti-like virus 24, and Lonestar tick totivirus—formed clades with their respective Chinese counterparts [28], suggesting either a common origin or regional adaptation.

Our novel isolate Qingyuan parti tick virus 1, a member of the *Partitiviridae* family, was detected for the first time outside of China in *H. asiaticum*. As only one reference strain was available in databases, a full phylogenetic tree could not be constructed. This marks the first confirmed detection of this virus outside China and underscores the need for continued molecular surveillance of Qingyuan parti tick virus 1 across Central Asia.

Wad Medani virus is one of the few tick-borne viruses known since the 1950s. It was first identified in Sudan from *Rhipicephalus sanguineus* [34] and later found in *H. asiaticum* and *H. anatolicum* in Central Asia, including Kazakhstan [7]. The new Kazakhstan Wad Medani virus isolate clustered with a Russian strain identified in *H. asiaticum* in 1985.

Bole tick virus 1 and Bole tick virus 3 were first isolated from *H. asiaticum* in China. Notably, Bole tick virus 1 has also been detected in humans, camels, and rodents [28], indicating a broad host range and potential zoonotic risk. The new Kazakhstan isolate of Bole tick virus 1 formed a distinct subclade, suggesting the emergence of a separate viral lineage in the region. Kazakhstan isolates of Bole tick virus 3 and Bole tick virus 4 showed close relatedness to Chinese strains but occupied distinct phylogenetic positions.

The obtained results demonstrated high sensitivity of the metagenomic approach to detection of viruses previously undescribed in Kazakhstan in *H. asiaticum* populations. Among the ten RNA viruses identified in this work, nine had not previously been registered in Kazakhstan. The fact of their presence, including viruses first discovered in China—Bole tick virus 1 (family *Phenuiviridae*), Bole tick virus 3 (*Chuviridae*), Bole tick virus 4 (*Flaviviridae*), Hubei toti-like virus 24 (*Totiviridae*), Kashgar totiv tick virus 1 (*Totiviridae*), Lonestar tick totivirus (*Totiviridae*), Qingyuan parti tick virus 1 (*Partitiviridae*), and Taishun tick virus (*Rhabdoviridae*)—indicates not only the possible transboundary circulation of viruses but also a significant underestimation of the biodiversity in the viral communities of ticks in the region. In Kazakhstan, *H. asiaticum* ticks act as important reservoirs of viruses [35], many of which may not be detected using classical methods. The viruses studied were found exclusively in ticks; the Wad Medani virus can infect vertebrates, such as laboratory mice. Tamdy virus belongs to a group of viruses that include those dangerous to humans and animals, so it can potentially infect vertebrates. The remaining viruses—Taishun tick virus, Bole tick virus 1, Bole tick virus 3, Bole tick virus 4, Hubei toti-like virus 24, Kashgar totiv tick virus 1, Lonestar tick totivirus, and Qingyuan parti tick virus 1—have so far only been found in ticks, and there are no data on their ability to infect vertebrates.

The data suggest evidence of cross-border viral circulation and local viral evolution. The detection of viruses from the families *Flaviviridae, Totiviridae, Phenuiviridae, Rhabdoviridae, Chuviridae, Sedoreoviridae*, and *Partitiviridae* in southern Kazakhstan highlights the high diversity of viral populations circulating in *H. asiaticum*. These findings emphasize the need for ongoing molecular surveillance and risk assessment regarding their potential threat to humans and animals.

According to FAO data, over 80% of the global cattle population is infested with ticks, resulting in significant economic losses, including reduced productivity, skin defects, animal illness, and mortality [36].

Over the past two decades, the distribution of ticks and tick-borne pathogens has rapidly changed, with expansions into new regions. This shift has been driven by various ecological and socio-economic factors, including climate change [37]. Environmental changes have promoted tick spread into new geographical areas that have since become epicenters of tick-borne diseases. According to UN reports, Kazakhstan’s climate is warming: the average annual temperature increased by 0.9 °C in 1991–2020 compared to 1961–1990. The year 2024 was among the most anomalously hot in Kazakhstan’s climate history. Such climate change may lead to range shifts in tick distribution. Tick-borne diseases have been recognized as a significant public health threat in Kazakhstan [38,39]. The global spread of ticks and the emergence of diverse tick-borne viruses suggest a growing risk of novel tick-borne diseases [40,41,42]. These factors all highlight the importance of focusing on tick surveillance, disease transmission, treatment, and prevention.

Modern metagenomic approaches, including next-generation sequencing (NGS) [43,44], enable direct identification of both known and novel pathogens—including viruses, bacteria, fungi, and parasites—from clinical or environmental samples [45,46,47]. These methods are especially valuable in cases of unclear etiology, mixed infections, or immunosuppression. Thus, metagenomic analysis has become a critical tool for studying the circulation of emerging pathogens and assessing their potential risks [48,49].

This study is the first metagenomic study of *H. asiaticum*-associated RNA viruses conducted in Kazakhstan and provides new data on the regional diversity of viruses previously undescribed in the country. The results highlight the need for regular epidemiological monitoring, as their potential impact on human and animal health remains poorly understood.

## 5. Conclusions

The results of this study revealed notable viral diversity associated with *H. asiaticum* in the southern regions of Kazakhstan. Using metagenomic analysis, nine RNA-containing viruses belonging to seven families were identified for the first time in this region: Bole tick virus 1 (*Phenuiviridae*), Bole tick virus 3 (*Chuviridae*), Bole tick virus 4 (*Flaviviridae*), Hubei toti-like virus 24 (Totiviridae), Kashgar totiv tick virus 1 (*Totiviridae*), Lonestar tick totivirus (*Totiviridae*), Qingyuan parti tick virus 1 (*Partitiviridae*), Taishun tick virus (*Rhabdoviridae*), and Wad Medani virus (*Sedoreoviridae*). These viruses, now circulating in Kazakhstan, were previously described in China. Their detection lays the groundwork for further in-depth research and improved surveillance of tick-borne viral diseases in the region.

## Figures and Tables

**Table 1:** Description of *H. asiaticum* samples from southern Kazakhstan.

Genbank Accession No.	Tick Species	Sampling Site	Location	Number of Ticks	Sex	Host
PV688046	*H. asiaticum*	Kyzylorda_ Muratbayev	45°04′55″ N, 64°40′51″ E	45 (3 pools × 15 ticks)	Female	Cattle
PV687729	*H. asiaticum*	Zhambyl_ Kulan	42°54′37″ N, 72°42′21″ E	45 (3 pools × 15 ticks)	Female	Cattle
PV687724	*H. asiaticum*	Turkestan_ Darbaza	41°34′06″ N, 69°04′59″ E	45 (3 pools × 15 ticks)	Female	Cattle

## Data Availability

The data presented in this study are available on request from the corresponding author.
